# Is meningococcal B vaccination needed in the post-COVID-19 world?

**DOI:** 10.7705/biomedica.7423

**Published:** 2025-08-11

**Authors:** Amanda Izeli Portilho, Elizabeth De Gaspari

**Affiliations:** 1 Centro de Imunologia do Instituto Adolfo Lutz, São Paulo, SP, Brasil Centro de Imunologia do Instituto Adolfo Lutz Centro de Imunologia do Instituto Adolfo Lutz São Paulo, SP Brasil; 2 Programa de Pós-Graduação Interunidades em Biotecnologia, Universidade de São Paulo, São Paulo, SP, Brasil Universidade de São Paulo Universidade de São Paulo São Paulo, SP Brasil

**Keywords:** Neisseria meningitidis, meningococcal disease, respiratory infections, coronavirus infections., Neisseria meningitidis, infecciones meningocócicas, infecciones del sistema respiratorio, infecciones por coronavirus.

## Abstract

*Neisseria meningitidis* is the main cause of bacterial meningitis worldwide and is transmitted through respiratory secretions. Meningitis is a serious public health problem because of its high morbidity and mortality rates and the risk of causing epidemics.

Although vaccines are available to prevent meningococcal disease, serogroup B infections are still challenging, given that many countries do not include meningococcal B vaccines in their national immunization programs. In addition, recent data suggests somewhat sustained *N. meningitidis* B infections during the COVID-19 pandemic and increasing levels of meningococcal disease after its control. These findings agree with previous observations indicating that respiratory viruses facilitate respiratory bacterial infections.

This essay intends to present epidemiological data on meningococcal disease and discusses studies exploring why the prevention of bacterial and viral infections is an intricate subject.

## Resumen

*Neisseria meningitidis* es la principal causa de meningitis bacteriana en el mundo y es transmitida mediante secreciones respiratorias. La meningitis es un grave problema de salud pública debido a sus grandes tasas de morbilidad y mortalidad, y por el riesgo de generar epidemias.

Aunque existen vacunas para prevenir la enfermedad meningocócica, la infección por el serogrupo B sigue siendo un desafío, ya que muchos países no han incluido estas vacunas en sus programas nacionales de inmunización. Además, datos recientes sugieren la persistencia de infecciones causadas por el serogrupo B de *N. meningitidis* durante la pandemia de COVID-19 y un incremento en los niveles de enfermedad meningocócica tras su contención. Esta información coincide con observaciones previas que afirman que los virus respiratorios facilitan las infecciones bacterianas respiratorias.

Este ensayo pretende presentar datos epidemiológicos de la enfermedad meningocócica y discutir aquellos estudios que resaltan la complejidad de la prevención de las infecciones bacterianas y virales.

Meningococcal disease is caused by the Gram-negative bacterium *Neisseria meningitidis*, which is classified into serogroups based on the polysaccharide composition of its capsule. Six serogroups (A, B, C, W, X, and Y) are mainly associated with invasive disease, and the bacterium is a strictly human pathogen transmitted via respiratory secretions ^1^. The seroprevalence of meningococcal strains is dynamic, changing according to time and location. Over the last decade, serogroup B has been primarily responsible for meningococcal disease in North and South America, Europe, Australia, New Zealand, South Africa, and other countries in Asia and the Middle East ^2^.

*Neisseria meningitidis* colonization is mediated by adhesins, such as type IV pili and opacity proteins (Opa and Opc). The progression to invasive disease involves bacterial dissemination through the bloodstream, where the capsule protects the bacteria against phagocytosis and antimicrobial peptides. Pathogenic strains are usually encapsulated, while carrier strains are not ^3^^,^^4^. The capsule is also important for host immunity, as vaccines against serogroups A, C, W, and Y are polysaccharide-conjugated. Their mechanism of protection relies on inducing bactericidal antibodies, which opsonize the bacteria and promote complement-mediated lysis ^1^.

The same approach was unsuitable for *N. meningitidis* serogroup B, a poor immunogen. Polysaccharide, a polymer of (α2-8)-linked sialic acid, is similar to human glycoproteins, especially, the neural cell adhesion molecules. These molecules are highly sialylated during fetal and neonatal stages, leading to cross-reactivity with meningococcal B polysaccharide. Besides poor immunogenicity, serogroup B polysaccharide vaccines could elicit autoimmune reactions ^5^^,^^6^.

As an alternative, researchers began studying protective subcapsular protein antigens, and in the 1980s, they developed outer membrane vesicle vaccines to control epidemics caused by *N. meningitidis* serogroup B. Later on, proteins of *N. meningitidis* were selected to create meningococcal B recombinant vaccines, currently approved in several countries: MenB-fHbp (Trumemba^®^, Pfizer), containing two variants of the factor H-binding protein (fHbp); and 4CMenB (Bexsero^®^, GlaxoSmithKline), composed of *Neisseria* adhesin A (NadA), *Neisseria* heparin-binding antigen (NHBA), fHbp, and outer membrane vesicles from an epidemic strain ^1^.

## Meningococcal disease incidence during and after COVID-19 pandemic

**Figure 1.**
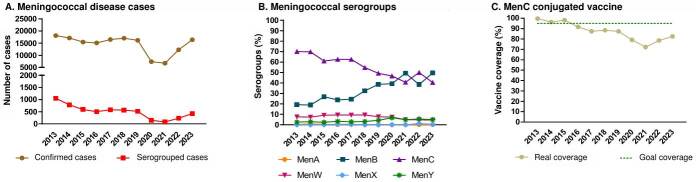
Meningococcal disease overview in Brazil between 2013-2023. A) The beginning of the COVID-19 pandemic, in 2020, revealed a decreased incidence, possibly due to social distancing and non-pharmacological policies to reduce SARS-CoV-2 transmission. However, since 2021, an increasing trend has been observed. B) The main serogroups circulating in Brazil are B and C. C) Meningococcal vaccination coverage was below the 95% goal even before COVID-19 in Brazil, but it reached its lowest point in 2021. The graph was elaborated using data from the official website of the *Departamento de Informática do Sistema Único de Saúde* (DATASUS), part of the Brazilian Ministry of Health.

During the COVID-19 pandemic, the incidence of meningococcal disease-and other bacterial infections transmitted through respiratory routes-decreased in most countries because of social distancing and other non-pharmaceutical strategies to control SARS-CoV-2 spread ^7^. However, meningococcal disease levels resurged once such measures were loosened ^8^. Nevertheless, the Secretary of Health of São Paulo state, in Brazil, reported a significant rise in meningococcal disease cases since 2022 ^9^, based on information compiled from the Brazilian Ministry of Health database. In the years preceding the pandemic, reported cases had declined, followed by an increasing trend in serogrouped and non-serogrouped cases. Among serogrouped cases, strains were mainly B and C (figure 1).

In France, meningococcal disease levels decreased during 2020 but began to increase in May 2021, with serogroup B contributing notably to this rise ^10^. Meanwhile, Israel reported a reduction in meningococcal disease incidence from 2020 to 2022; however, serogroup B strains were the most frequently isolated, particularly among children ^11^. In the Netherlands, while cases of meningococcal disease caused by serogroups W and Y declined by approximately 90% during the COVID-19 pandemic, serogroup B declined by 60% ^12^. Throughout 2020, England documented a reduction in meningococcal disease cases, although, in 2021, *N. meningitidis* serogroup B infections were particularly prevalent in adolescents, and in 2022-2023, the meningococcal disease reached its highest incidence since 2012 ^13^. Finally, a consortium of 30 countries recently published that meningococcal cases caused by serogroups C, W, and Y decreased during the COVID-19 pandemic, while serogroup B accounted for most cases ^8^. In the following section, we will discuss factors that could explain this increased incidence.

## Bacterial and viral respiratory infections alongside

Meningococcal B epidemics may show a gradual increase and often take years to return to endemic levels. Thus, adequate surveillance is required to identify epidemic trends as soon as possible ^14^^,^^15^. This aspect is particularly relevant after the COVID-19 pandemic, as the scientific community has discussed whether respiratory viral infections could facilitate bacterial diseases, mostly taking influenza as an object of study ^16^.

Current research about bacteria and COVID-19 co-infection is heterogeneous. A meta-analysis conducted in 2020 pointed to the predominance of *Mycoplasma pneumoniae, Haemophilus influenzae, and Pseudomonas aeruginosa*^7^. An article from 2023 supported such findings but added *Staphylococcus aureus and Streptococcus pneumoniae* to the list ^17^. Moreover, an interesting study found that, even though invasive disease caused by *S. pneumoniae* was reduced during the COVID-19 pandemic, carriage levels did not change ^18^.

Only a few manuscripts addressed COVID-19 and meningococcal disease co-infection. In 2020, Gallacher *and collaborators*^19^ described the case of a healthy 22-year-old woman co-infected with meningococcus serogroup B and SARS-CoV-2 but recovered without any sequelae. Notably, she had previously received meningococcal C and ACWY conjugate vaccines. According to this finding, the authors highlighted the importance of characterizing bacterial infections to avoid complications in COVID-19 patients.

Two additional cases attributed to *N. meningitidis* serogroup B had unconventional clinical presentations. The first was a case of meningococcal arthritis in an 18-year-old without relevant medical history, despite some evidence of autoimmune disease in relatives ^20^. The second case involved an elderly patient with comorbidities, including diabetes mellitus, hypertension, and airway disease comorbidities, who presented COVID-19 and meningococcal pneumonia-an even rarer manifestation of meningococcal disease. Despite hypoxia and lung involvement, the patient recovered well. Even though serogrouping was not performed, the authors suspected serogroup B according to the strain’s antibiotic resistance profile ^21^.

A pediatric case of a 7-year-old co-infected with SARS-CoV-2 and *N. meningitidis* serogroup C resulted in death. It happened in México, where the overall incidence of meningococcal disease is low, though no vaccines are included in the national immunization program. The patient had no clinically relevant history and was not vaccinated ^22^.

None of the previous reports provided a clear link between *Neisseria* and SARS-CoV-2. In Ducatez *et al.*^20^, the meningococcal arthritis occurred following COVID-19 and remained localized. The authors suggested that the viral infection may have rendered the nasal epithelium, facilitating dissemination of *N. meningitidis*, which was carried asymptomatically before.

When considering other respiratory viral infections, influenza A has been linked to anticipated meningococcal disease outbreaks in several countries ^23^. Rameix-Welti *et al.* found that viral neuraminidase enhances *N. meningitidis* adhesion to infected cells, a mechanism that facilitates bacterial attachment and invasion, which may help explain the increased incidence of meningococcal disease following influenza ^24^. Respiratory syncytial virus (RSV) was also implicated in increasing meningococcal disease incidence in Canada ^25^, but no mechanism explained it.

Despite the limited availability of mechanistic studies establishing any relationship between SARS-CoV-2 and *N. meningitidis* (or other bacteria), some evidence allows us to speculate how one may affect the other. Genomic analyses have shown that COVID-19 patients had nasopharyngeal dysbiosis, characterized by a less diverse microbiome and the presence of opportunistic bacteria ^26^. When comparing patients with low or high cycle threshold (Ct) values for SARS-CoV-2 molecular testing -where a low Ct in RT-qPCR indicates a higher viral load-, patients with a low Ct had more *Neisseria* and *Pseudomonas* species in their nasal microbiome ^27^. In the same study, the transcriptome of COVID-19 patients indicated downregulation of genes involved in ciliary function, cell-to-cell adhesion, and mucin production, suggesting impaired natural defenses of the upper airways ^27^. Disruption of epithelial organization and cilium shortening were confirmed *in vitro* using a model of pseudostratified airway epithelium derived from donor tracheobronchial cells ^28^.

Another study confirmed compromised mucociliary clearance and downregulation of occluding and claudin, proteins implicated in the formation of tight junctions, which facilitated *P. aeruginosa* penetration in a model of human bronchial epithelial cell lineage. These effects were attributed to the SARS-CoV-2 envelope (E) protein ^29^. If the natural defenses of the respiratory epithelium are impaired, bacteria may dominate the colonization site and encounter reduced resistance to adhesion and translocation, thereby favoring infection.

*Neisseria meningitidis* uses CD147 and carcinoembryonic antigen-related cell adhesion molecules (CEACAM) as receptors for type IV pili and opacity proteins, respectively ^4^. Viral infections, especially by SARS-CoV-2, have been shown to increase CEACAM1 expression in polymorphonuclear cells from human donors. Similar results were reported for CEACAM7 expression in human bronchial epithelial cells infected with the virus ^30^^,^^31^. If such upregulation occurred *in vivo*, it may enhance *Neisseria* interaction with its receptor and promote intimate adhesion to the epithelium, thus increasing the risk of meningococcal disease. Furthermore, an interesting study found that the spike of SARS-CoV-2 binds to lipopolysaccharides (LPS) expressed by Gram-negative bacteria. Co-administration of lipopolysaccharides and spike resulted in a more pronounced inflammatory response via NF-κβ ^32^. Although this finding does not prove a higher risk of developing meningococcal disease following SARS-CoV-2 infection, it suggests that a co-infection may exacerbate disease severity by uncontrolled inflammation.

## Pandemic impact on routine vaccination

During the COVID-19 pandemic, SARS-CoV-2 vaccines became the focus of interest. However, routine immunization was generally compromised. Several factors explained this situation: the tendency to avoid healthcare routine appointments for non-COVID-19 issues, pressure to manufacture SARS-CoV-2 vaccines resulting in a setback for other immunobiologicals, and reduced vaccine uptake because of misinformation propagated by antivaccine campaigns ^33^.

In Brazil, certain vaccine coverages faced relevant impacts in 2020 compared to previous years. For instance, vaccination against meningococcal serogroup C decreased by 25.02%, while coverage for influenza and diphtheria-pertussis-tetanus increased. Nonetheless, vaccine coverage for children under 10 years dropped from 77.12% in 2019 to 68% in 2020 ^34^.

Regarding children and maternal vaccination, a systematic review found that 17 out of 18 studies reported reduced coverage for many pediatric vaccines, such as bacillus Calmette-Guérin (BCG), hepatitis B, measles- mumps-rubella, diphtheria, and polio. These effects were more dramatic in low and middle-income countries: while high-income countries reported coverage reductions from -1.8% to -14%, the poorest countries faced reductions between -3.7% and -24%. Fewer studies assessed maternal immunization and also reported coverage decreases, resulting in reduced neonatal protection ^35^.

The Brazilian National Immunization Program has offered the meningococcal C- conjugate vaccine for infants since 2010, and the current goal is to reach 95% vaccine coverage. Although the coverage percentage has remained below ideal since 2016, its lowest point was in 2021 during the COVID-19 pandemic. Even though the coverage increased in the following years, it continued below the 95% goal (figure 1).

## Should we consider prevention strategies?

Current meningococcal B vaccines-Trumemba^®^ and Bexsero^®^-are effective and approved for use in 58 countries. However, only 24 have included them in their national immunization programs. Beyond the epidemiological context, the cost of these vaccines is a contributing factor in this decision ^36^. In Brazil, for example, serogroup B is one of the main isolated strains, but the national immunization program only offers C-conjugate vaccines for infants and ACWY-conjugate vaccines for adolescents ^37^. Further strategies are essential to provide meningococcal B vaccines nationwide ^36^. The potential benefit could be exemplified by data from England, where children eligible for meningococcal B vaccination accounted for fewer cases than other age groups during the recent peak of meningococcal disease ^13^.

Real-world evidence also supports the use of meningococcal B vaccines. The United Kingdom was the first country to adopt the 4CMenB vaccine (Bexsero^®^, GlaxoSmithKline) for children into its national immunization program, and they observed a 75% decrease in meningococcal disease cases among the vaccinated age group ^38^. In Italy, the vaccine efficacy was over 90% ^39^. Australia introduced the vaccine for infants and adolescents, and the respective efficacy was 63% and 78% in each group ^40^. In addition, this vaccine is likely to induce protection from other *N. meningitidis* strains besides the serogroup B and gonorrhea ^40^^,^^41^. MenB-fHbp (Trumemba^®^, Pfizer) is approved for people aged 10 years or older. It was efficacious in adolescents and adults ^42^^,^^43^. However, data about its use in infants and young children -who are more at high risk for meningococcal disease-are limited. Although information regarding its implementation in nationwide programs is also lacking, this vaccine was successful in controlling outbreaks ^44^.

Controlling the carriage of *N. meningitidis* in the nasopharynx would be another relevant aspect of meningococcal disease prevention. Polysaccharide-conjugate vaccines (which protect against serogroups A, C, W, and Y) effectively reduce meningococcal carriage, but conflicting results were observed for meningococcal B vaccines. In the United Kingdom, a study among university students exhibited reduced meningococcal carrier strains from serogroups B, C, W, and Y, three months after vaccination with 4CMenB ^45^. For Australian adolescents (between 15 and 18 years old), vaccination did not reduce the nasopharyngeal density of *N. meningitidis* significantly ^46^. Likewise, MenB-fHbp did not seem to reduce meningococcal carriage following a college outbreak ^44^.

The evidence described above shows the impact of meningococcal B vaccines, however, a common aspect is that such investigations were conducted in high-income countries. Modeling studies concluded that 4CMenB would not be cost-effective for inclusion in the Brazilian National Immunization Program if the cost per dose was equivalent to that in European countries. Meanwhile, for an outbreak situation in Chile, 4CMenB would only be cost-effective if the price per dose were below USD 18 ^47^^,^^48^. Even in the United States, a high-income country, meningococcal B vaccination coverage was suboptimal in counties with lower socioeconomic status ^49^. All that considered, the economic burden of implementing recombinant meningococcal B vaccines in government-funded national programs remains a challenge to control *N. meningitidis* serogroup B.

Apart from economic constraints, immunization is required to control infectious diseases. Therefore, researchers should explore strategies to reach the full potential of available vaccines. George *et al.* discussed that vaccines are likely to affect one another; for example, influenza infection has preceded meningococcal disease peaks. This finding could suggest that the population would benefit from a combined policy for immunization. Thus, further studies to comprehend such dynamics should be encouraged ^50^. Similarly, Bloom *et al.* not only advocate for meningococcal immunization following COVID-19, but highlight its urgency due to the gaps in routine vaccination after the pandemic ^51^.

## Conclusion

After facing the COVID-19 pandemic, when the anti-vaccine movements were remarkably vocal, the scientific community should engage in initiatives to raise public awareness about the safety and efficacy of vaccines. In the case of meningococcal infections, because of the rapid evolution and high morbidity and mortality rates, vaccines are the most effective tool to control the disease. Here, we suggest that meningococcal B vaccination should be addressed considering the post-SARS-CoV-2 scenario and the increase in meningococcal disease cases. Reduced vaccine coverage after the COVID-19 pandemic and the high cost of implementation are relevant challenges, especially for low- and middle-income countries. If we consider the ongoing threat of emerging viruses, the prevention of bacterial and viral respiratory infections could benefit from an integrated approach.

Future strategies should involve searching for new, multi-pathogen vaccine preparations, aiming affordable technologies for low- and middle- income countries; vaccinating pregnant women (to protect newborns from meningococcal disease) and the elderly (susceptible to respiratory infections); and evaluating policy changes-as annual respiratory-pathogens catch-up campaigns-to enhance vaccine coverage.
